# The prevalence of *Escherichia coli* O157:H7 fecal shedding in feedlot pens is affected by the water-to-cattle ratio: A randomized controlled trial

**DOI:** 10.1371/journal.pone.0192149

**Published:** 2018-02-07

**Authors:** Wendy Beauvais, Elena V. Gart, Melissa Bean, Anthony Blanco, Jennifer Wilsey, Kallie McWhinney, Laura Bryan, Mary Krath, Ching-Yuan Yang, Diego Manriquez Alvarez, Sushil Paudyal, Kelsey Bryan, Samantha Stewart, Peter W. Cook, Glenn Lahodny, Karina Baumgarten, Raju Gautam, Kendra Nightingale, Sara D. Lawhon, Pablo Pinedo, Renata Ivanek

**Affiliations:** 1 College of Veterinary Medicine, Cornell University, Ithaca, New York, United States of America; 2 College of Veterinary Medicine and Biomedical Sciences, Texas A&M University, College Station, Texas, United States of America; 3 West Texas A&M University, Canyon, Texas, United States of America; 4 Texas A&M Agrilife Research, Amarillo, Texas, United States of America; 5 Animal and Food Sciences, Texas Tech University, Lubbock, Texas, United States of America; ContraFect Corporation, UNITED STATES

## Abstract

*Escherichia coli* O157:H7 fecal shedding in feedlot cattle is common and is a public health concern due to the risk of foodborne transmission that can result in severe, or even fatal, disease in people. Despite a large body of research, few practical and cost-effective farm-level interventions have been identified. In this study, a randomized controlled trial was conducted to assess the effect of reducing the level of water in automatically refilling water-troughs on fecal shedding of *E*. *coli* O157:H7 in feedlot cattle. Pens in a feedlot in the Texas Panhandle were randomly allocated as control (total number: 17) or intervention (total number: 18) pens. Fecal samples (2,759 in total) were collected both at baseline and three weeks after the intervention, and tested for the presence of *E*. *coli* O157:H7 using immunomagnetic bead separation and selective culture. There was a strong statistical association between sampling date and the likelihood of a fecal sample testing positive for *E*. *coli* O157:H7. Pen was also a strong predictor of fecal prevalence. Despite accounting for this high level of clustering, a statistically significant association between reduced water levels in the trough and increased prevalence of *E*. *coli* O157:H7 in the feces was observed (Odds Ratio = 1.6; 95% Confidence Interval: 1.2–2.0; Likelihood Ratio Test: p = 0.02). This is the first time that such an association has been reported, and suggests that increasing water-trough levels may be effective in reducing shedding of *E*. *coli* O157:H7 in cattle feces, although further work would be needed to test this hypothesis. Controlling *E*. *coli* O157:H7 fecal shedding at the pre-harvest level may lead to a reduced burden of human foodborne illness attributed to this pathogen in beef.

## Introduction

*Escherichia coli* O157:H7 is the predominant serotype representing the Shiga-toxin producing *E*. *coli* group associated with human disease. It was first identified as a pathogen in 1982 during an outbreak investigation of hemorrhagic colitis [1]. Human infections caused by *E*. *coli* O157:H7 result in clinical syndromes ranging from asymptomatic to severe [2]. A few individuals may develop potentially fatal complications such as hemolytic uremic syndrome [3]. A chronic renal sequela may persist among those that survive [4].

The Centers for Disease Control and Prevention have estimated that in the United States, foodborne *E*. *coli* O157:H7 causes over 63,000 illnesses per year resulting in more than 2,100 hospitalizations and 20 deaths [5]. The total annual economic burden of illness due to *E*. *coli* O157:H7 resulting from medical expenses and loss of life and productivity is estimated to be $405 million [6]. The beef industry suffers economic losses directly from recalls of *E*. *coli* O157:H7-contaminated raw non-intact beef (and its components) and indirectly from subsequent decline in consumer demand for beef in the market. *E*. *coli* O157:H7 was responsible for the highest number of beef recalls between 1982–1999 [7]. It has been reported that beef recalls by the Food Safety Inspection Services caused a decline in beef demand [8]. Consumer buying practices are greatly influenced by perception of the food safety of any given product [9, 10].

There are multiple ways by which humans are known to acquire an *E*. *coli* O157:H7 infection, including contaminated food, water, and direct contact with infected animals and humans [11–15]. The majority of human outbreaks and illnesses, however, have been linked to consumption of contaminated food including ground beef, dairy products, and fresh produce [16]. Cattle are the principal reservoir of *E*. *coli* O157:H7 for human infections [17]. Cattle that are colonized by and shedding *E*. *coli* O157:H7 are not only the source of food contamination at slaughter, but their manure may also contaminate fruits and vegetables when used as fertilizer [18]. Furthermore, water supplies may be contaminated by runoff from cattle farms which may cause human illness [19]. Many human outbreaks of *E*. *coli* O157:H7 infections associated with fresh produce and water have been directly linked to cattle operations [20].

Published literature has reported great variation in the prevalence of *E*. *coli* O157:H7 fecal shedding, with estimates ranging from 0 to 80% among cattle populations [21, 22]. Fecal shedding of *E*. *coli* O157:H7 has a seasonal pattern, with prevalence rising to a peak during the summer months and declining in the winter months [23]. The elevated temperature in the summer seems to provide favorable conditions for the bacteria to survive and potentially even multiply to increase numbers in the operation environment [24–27]. Variability in shedding prevalence has also been observed between different pens within a feedlot at a single time-point, and over time [22]. This variability offers an opportunity to carefully examine environmental and farm management practices as potential driving forces and to identify new targets for an effective control strategy to reduce the shedding prevalence of *E*. *coli* O157:H7 in cattle.

A considerably large body of research efforts has been directed towards reducing fecal shedding of *E*. *coli* O157:H7 in cattle over recent years. Many intervention strategies have been suggested, such as the use of probiotics, antibiotics, vaccinations, bacteriophages, dietary changes, and improved animal management [19]. However, these interventions have been shown to have either a weak effect, or the observed effect lacks repeatability when evaluated against naturally colonized cattle under field conditions. They may also be logistically difficult to implement, sometimes even failing to motivate producers to adopt them [28,29].

A survey of selected feed yards in the Texas Panhandle indicated that feedlot pens vary with respect to the water-to-cattle ratios, ranging between 0.4 and 4.5 (median 0.9) liters per head [30]. Water-to-cattle ratio denotes the amount of standing water at a point in time available per head of cattle; it is estimated by dividing the volume of water in water-troughs in a pen with the number of animals in the pen. Water-troughs have an automatic water refill system that assures water refilling as cattle drink. Prior mathematical modelling studies [24, 31] suggest that if the water trough volume is high relative to the number of animals in the pen (high water-to-cattle ratio), a large pool of standing water is available, which in warm weather will heat up and may promote *E*. *coli* O157:H7 growth. Thus, drinking water was identified as a potential pathway of infection transmission with drinking water-to-cattle ratio as a strong modulator of the process. This gave rise to a hypothesis that at a given elevated ambient temperature, a lowered water-to-cattle ratio will secure a faster refill of fresh water and reduce the growth and accumulation of *E*. *coli* O157:H7 in water, leading to a reduced prevalence and load of fecal shedding among cattle. The primary objective of this study was to test this hypothesis by conducting a controlled intervention trial to assess the effectiveness of reducing drinking water-to-cattle ratio as a strategy to reduce *E*. *coli* O157:H7 fecal shedding prevalence in feedlot cattle. The second objective was to quantify the variability in *E*. *coli* O157:H7 fecal shedding prevalence within and between pens under controlled conditions and identify predictors that explain this variability.

## Materials and methods

### Overview

A randomized controlled trial was conducted between 16^th^ June and 21^st^ July in 2014, and repeated between 16^th^ June and 20^th^ July in 2015. Pens within a single feedlot, that was previously identified as positive for *E*. *coli* O157:H7, were first screened for the presence of *E*. *coli* O157:H7 and then a subset of positive pens was randomly allocated to either intervention group (water-trough filling level reduced by 50% of the baseline level, which was a near approximation to a 50% reduction in water volume) or control group (no change to the water-trough level). Water-trough dimensions varied among pens. It should be noted that because of automatic refilling, the intervention had no effect on the free access of cattle to water. Fecal samples were collected both at baseline and three weeks after the intervention, and tested for the presence *E*. *coli* O157:H7. Also, water samples were collected from each pen trough both pre- and post-intervention and tested for the presence of *E*. *coli* O157:H7, generic *E*. *coli* and coliforms. The three-week re-testing period was based on the longest length of *E*. *coli* O157:H7 colonization in an experimental infection [32] and predictions from an unpublished feedlot adaptation of a mathematical model [24]. For logistical reasons, the pens were assigned to three different cohorts each year, which started 1 week apart from each other. The Texas A&M University’s Institutional Animal Care and Use Committee (IACUC) evaluated the study and determined that it did not require an Animal Use Protocol because it did not involve the handling or manipulation of live animals to obtain samples for research and did not involve altering the lifestyle of the animals for research purposes (decision letter dated October 13, 2013).

### Study farm

A feedlot in the Texas Panhandle, a major beef production region, was selected for the study as it was considered a typical feedlot and had *E*. *coli* O157:H7 detected from fecal samples prior to this study. In 2014, the feedlot housed approximately 50,000 Holstein steers from post weaning age up to 390 days in feeding (days since arrival on the feedlot) in 192 pens. In 2015, out of 203 stocked pens, the number of heads and days in feeding were available only for the pens identified as positive.

### Environmental screening of pens for *E*. *coli* O157:H7

Environmental screening was conducted on May 30, 2014 and on June 4, 2015. All occupied pens (192 in 2014 and 203 in 2015) were screened for *E*. *coli* O157:H7 by collecting a single gauze swab (boot sock) sample from the floor of the pen and conducting selective culture and latex agglutination testing. In order to collect a representative sample from each pen, the swab was placed over a plastic boot cover near the ball and in-step areas of one foot and sprayed with distilled water if the pen floor was very dry. The individual in the pen was then instructed to walk along the feed trough near the edge of the pen. Gloves and plastic boot covers were changed between each pen. Once the person had walked approximately 100 feet, the swab was removed and placed into a specimen cup that contained 50 ml of gram negative broth containing vancomycin (40 μg/ml), cefsulodin (10 μg/ml) and cefixime (0.05 μg/ml). Specimen cups were kept in coolers or refrigerators until they were delivered to the microbiology laboratory at Texas A&M University within 24 hours where they were placed in an incubator at 37°C and left to enrich overnight. The next day the samples underwent immunomagnetic bead separation with 20 μl of Dynabeads suspension (anti-*E*. *coli* O157; Dynal Biotech ASA, Oslo, Norway). From the specimen cup, 1 ml of solution was removed and added to a microcentrifuge tube containing the beads. The samples then underwent immunomagnetic separation, selective culture and latex agglutination testing as described below.

### Allocation of control and intervention pens and cohorts

All pens were screened for the presence of *E*. *coli* O157:H7 in the pen environment as described above. In 2014 (year 1) 83% (19/ 23) of *E*. *coli* O157:H7 positive pens were included in the trial, while in 2015 (year 2) 46% (16 of 35) of positive pens were included in the trial (explained below). Pens were allocated to three different cohorts (1–3), beginning on June 16, June 23 and June 30 in 2014 and on June 16, June 23 and June 29 in 2015. Blocking was used in order to ensure that cohorts were similar with respect to days in feeding: pens were first ordered by days in feeding, then systematically randomly assigned to cohorts 1, 2 or 3 by allocating two successive pens (a control and intervention pen) to a different cohort.

### Adaptation to trial design

In year 2, several commercial field trials were also being conducted at the participating feedlot, which had started operating under new management. As a result, the pen selection and treatment assignment method used in our study excluded pens that were being used in the underlying commercial field trials when experimental groups and replicates could not be balanced in their commercial trials. The information about the nature of their trials was proprietary and was not shared with the authors. Out of the total of 35 *E*. *coli* O157:H7 positive pens, 9 were available for selection as intervention pens, of which 8 were selected. A pen that was 376 days in feeding was excluded because it was scheduled for slaughter. The remaining 26 positive pens were available for enrollment as control pens and 8 pens with the distribution of days in feeding similar to that for the intervention pens were allocated as control pens.

### Sample selection and transport to laboratory for processing

The following samples were collected from each pen at the start and end of the trial (three weeks later).

1. Forty individual well-formed, freshly voided fecal pats were randomly sampled from each pen. Using a disposable spoon, approximately 20 g of feces were collected per sample into Ziploc bags and shipped on ice overnight to Texas A&M University for processing within 24 hours.
2. One 200 ml water sample was collected from the center-point of the trough (mid-depth) in each pen into sterile plastic containers and shipped on ice overnight to the Texas Tech University for processing within 24 hours.

### Sample size calculation

The enrolled 18 intervention and 17 control pens (with 40 samples collected per pen) provided 80% power and 95% confidence to detect at least 15% difference in *E*. *coli* O157:H7 fecal shedding prevalence at the fecal pat level between the intervention and control pens. The calculation assumed the intra-class correlation of 0.005 based on the expected within-pen prevalence of 26% [33] and 100% between-pen prevalence considering that only positive pens were enrolled into the trial. The sample size calculation was performed using the “pwr” package in R [34].

### Laboratory tests

#### Fecal samples

Fecal samples were tested following post-enrichment direct streak as previously described [35]. Briefly, fecal samples were mixed for homogeneity and 1 gram of fecal material was placed into a test tube containing 9 ml of gram negative broth (Becton Dickinson Co., Franklin Lakes, NJ) containing vancomycin (8 μg/ml), cefsulodin (10 μg/ml) and cefixime (0.05 μg/ml) and incubated overnight at 37°C. Each sample was vortexed and then 1 ml of solution was placed into a microcentrifuge tube containing immunomagnetic beads (Dynabeads; anti-*E*. *coli* O157; Dynal Biotech ASA, Oslo, Norway) and *E*. *coli* O157:H7 was captured as described by the manufacturer’s protocol. The beads were mixed into the solution and washed three times using washing buffer with magnetic capture in between washes. All but 100 μl of the solution were removed after the final wash. Those 100 μl were then dropped, swabbed, and struck onto a sorbitol MacConkey agar plate containing 50 μl of cefixime (0.05 mg/l) and 50 μl of potassium tellurite (2.5 mg/l). The plates were then incubated overnight at 37°C. The plates were visually examined for colonies that appeared to be non-sorbitol-fermenting (gray) colonies at 24 hours and 48 hours post inoculation. A maximum of four of the non-sorbitol-fermenting colonies per plate were handpicked and struck onto blood agar plates. Plates without non-sorbitol-fermenting colonies were recorded as negative samples and did not undergo further testing. The blood agar plates were incubated overnight at 37°C. The next day colonies from the blood agar plates were tested using a spot indole test. Positive samples underwent confirmatory testing using the latex agglutination test (E. COLIPRO™ O157 KIT, Hardy Diagnostics). In year 2, *E*. *coli* O157:H7 in fecal samples were enumerated using a spiral plating approach, with PCR confirmation. Spiral plating was performed as previously described [35] using a commercially available spiral plating system (Eddy Jet 2- Spiral Plater, Neutec Group, Inc, Farmingdale, NY) according to the manufacturer’s instructions. Colonies were confirmed using the PCR primers rfbE-F 5’-CTGTCCACACGATGCCAATG-3’ and rfbE-R CGATAGGCTGGGGAAACTAGG-3’ and probe 5’-Fam-TTAATTCCACGCCAACCAAGATCCTCABHQ1-3’ [36]. PCR primers and probe were used at a final concentration of 500nM. The PCR mastermix was TaqMan Fast Universal PCR Mastermix (2X) (Applied Biosystems, ThermoFisher, Foster City, CA). PCR was performed using the 7900HT Fast Real Time PCR machine (Applied Biosystems) with the cycling parameters 20 seconds at 95°C followed by 40 cycles of 20 seconds at 60°C and 1 second at 95°C.

#### Water samples

A 1 ml aliquot from each water sample (approx. 200 ml) and 1 ml of a 1:10 dilution of each water sample were directly plated onto *E*. *coli*/coliform petrifilm. Petrifilm were incubated and enumerated to determine generic *E*. *coli* and coliforms counts/ml of water according to the manufacturer’s instructions (3M Technologies). A 100 ml aliquot of each water sample was passed through a sterile 0.45 uM bottle top filtration unit (Corning) using a vacuum. Each filter was aseptically transferred to a sterile whirl-pak bag, combined with mTSB and incubated at 42°C for 18–24 h. Enriched filters were screened for the presence of *E*. *coli* O157:H7 using the BAX® Real-Time PCR assay for *E*. *coli* O157:H7 according to the manufacturers’ instructions (Dupont, Qualicon). PCR screen positive enrichments were plated onto selective and differential media and up to four presumptive positive colonies were subjected to confirmation using a modified version of the USDA: FSIS Microbiology Laboratory Guide.

#### Sensitivity and specificity of the tests

The selective culture and latex agglutination testing, which was used in the environmental screening and testing of fecal samples, has been estimated to have a sensitivity and specificity of 86.1% and 86.7%, respectively, in detecting nalidixic acid (Nal)-resistant *E*. *coli* O157 on the agar supplemented with Nal [35]. The approach has been considered to be highly sensitive at concentrations above 100 CFU/g [35,37]. Without the confirmatory PCR test following the selective culture and latex agglutination testing, our approach was likely to have a slightly higher sensitivity but lower specificity compared to the sensitivity and specificity values reported in [35]. As we were interested in the effect of the intervention on the distribution of super-shedders, the limit of detection for spiral plating chosen for our study was 4 log_10_ CFU/g. At that detection limit, the reported sensitivity and specificity of spiral plating were 34.2% and 88.1%, respectively [35], meaning that this test is relatively good at correctly classifying those animals that are not super-shedders, albeit at the cost of a high probability of misclassifying super-shedding animals as false negatives. The limit of detection for the presence of *E*. *coli* O157:H7 in water samples was 1 CFU/100 ml of water, because 100ml of water was filtered prior to enrichment.

### Data collection

The following data were collected for each pen: number of cattle, mean days in feeding and volume of water in the water-trough. For each day of sample collection, the following meteorological data were obtained from the nearest weather station (approximately 50 km from the feedlot) via http://www.wunderground.com/history/:

- minimum, average and maximum temperatures over the previous seven days;
- minimum, average and maximum relative humidity over the previous seven days;
- minimum, average and maximum dew-point over the previous seven days; and
- cumulative rainfall over the previous seven days.

### Intervention

In intervention pens, the level of water in the trough was adjusted to 50% of the baseline level (i.e. the level that was maintained by the feedlot before the trial began) for the duration of the trial. All troughs in the trial had automated filling, which ensured that the trough water level remained consistent throughout the trial. In control pens, no adjustments were made to the level of water in the trough at baseline.

### Statistical analysis

**Fig 1:**
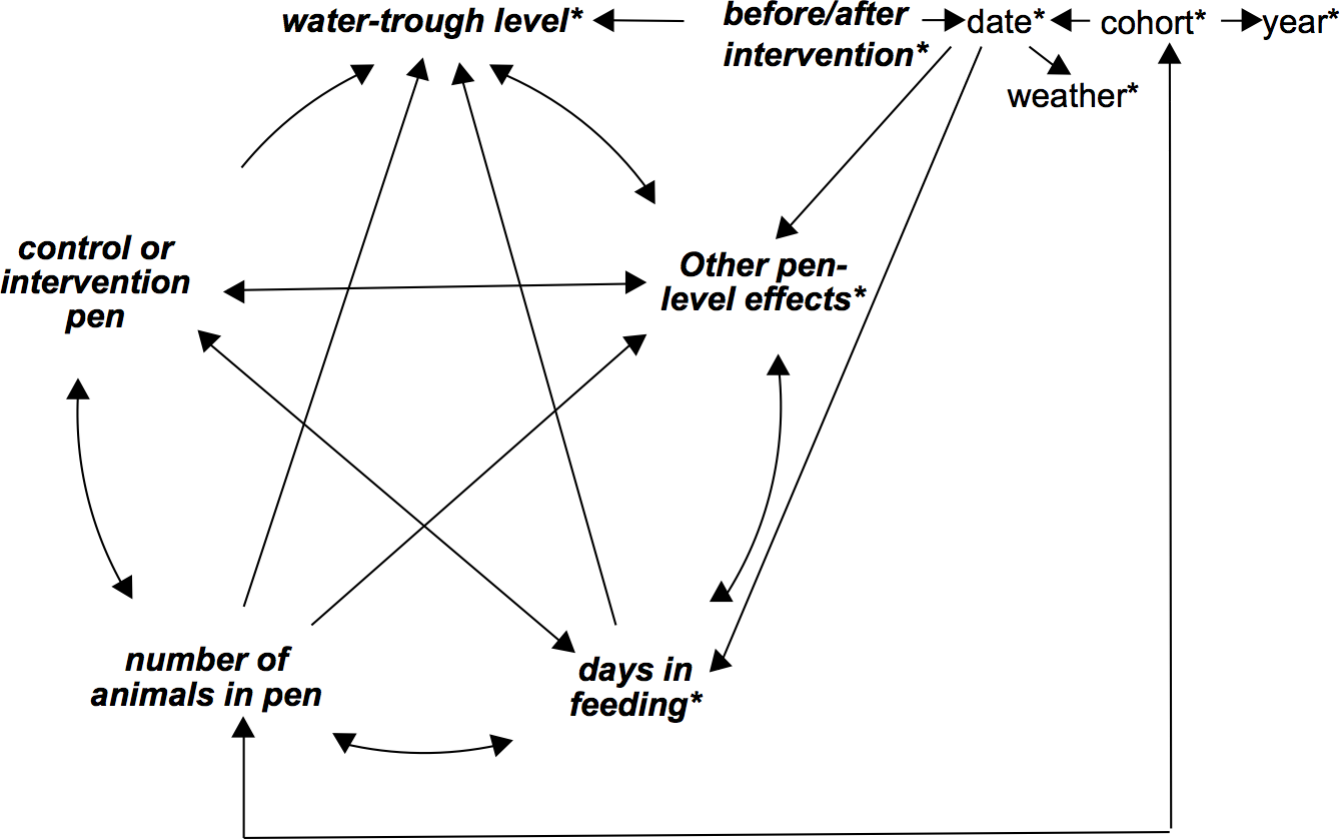
Putative causal diagram of factors that may influence the likelihood of *E*. *coli* O157:H7 in cattle feces. Pen-dependent factors are in bold and italic typeface. Time-dependent factors are marked with an *. Lines indicate a putative association between factors.

Data were entered into Microsoft Excel spreadsheets. Data checks, cleaning and statistical analyses were carried out in R [38]. Firstly, descriptive analyses were conducted. Secondly, potential risk factors for pen status (positive or negative), based on environmental screening, were explored (this analysis was possible for year 1 data only). Thirdly, the potential effects of the intervention (reducing water-trough level) were investigated. Although randomization was used to assign intervention status, and blocking was used to further mitigate the risk of confounding, the weather was variable during the study and known to be a potential predictor of *E*. *coli* O157:H7 prevalence. We therefore conducted a full risk factor analysis to identify potential confounders of the association between intervention status and *E*. *coli* O157:H7 prevalence. For this purpose, a putative causal diagram was constructed (Fig 1). Each factor was explored in the univariable and multivariable analyses, grouped by (i) weather variables, (ii) pen-level variables, and (iii) intervention, using a subset or whole dataset: the control pen data to evaluate the weather variables, the pre-intervention data to evaluate the pen-level variables, and the whole dataset to evaluate the intervention. Fourthly, the effects of these factors on counts of coliforms or *E*. *coli* in the water trough were explored. Fifth, the associations between counts of coliforms or *E*. *coli* in the water trough and the odds of an individual fecal pat being positive for *E*. *coli* O157:H7 were explored. Finally, we investigated the CFU/g of *E*. *coli O157*:*H7* in fecal samples (this analysis was possible for year 2 only). The correction for multiple comparisons in the univariate analyses was not conducted due to the exploratory nature of the investigation. For all multivariable analyses, a forward-selection stepwise procedure was followed, beginning with univariate screening of each variable. Variables were dropped from the multivariable models according to significance criteria (described in detail below).

#### Descriptive analysis

The proportions of pens, and of fecal samples (and 95% confidence interval) that were positive for *E*. *coli* O157:H7 were estimated. The latter estimate was weighted by the inverse of the sampling fraction within each pen using the ‘svy’ package in R [39]. Summary statistics and plots to describe the distribution of each potential explanatory variable were also produced: days in feeding, number of heads per pen and weather variables. These variables were compared with respect to intervention allocation group.

#### Risk factor analysis for contamination at pen-level

Pens identified as positive and negative for *E*. *coli* O157:H7 based on the environmental screening were compared with respect to days in feeding and number of animals in the pen, and the significance of any association was tested using the Mann-Whitney U Test.

#### Univariable analyses of associations between risk factors, intervention and proportion of fecal pats positive for *E*. *coli* O157:H7

Data from control pens only were used to assess the effect of weather on the prevalence of *E*. *coli* O157:H7 in fecal pats. Scatterplots were used to show the univariable relationships between prevalence on each sampling date and weather variables (temperature, humidity, dew-point and rainfall). Each weather variable was assessed separately. Separate logistic regression models were constructed to estimate the univariable effect of each weather variable. The variables were modelled both as being linearly associated with the log odds of *E*. *coli* O157:H7 in fecal pats and as 3-level categorical indicator variables (each level having equal numbers of data points). Diagnostic plots (observed versus predicted log odds of *E*. *coli* O157:H7 presence; Pearson residuals versus value of each pen-level variable and Pearson residuals versus predicted log odds of *E*. *coli* O157:H7) were used to assess the suitability of models that assume a linear relationship between each pen-level variable and the log odds of *E*. *coli* O157:H7 in fecal pats.

Data from the pre-intervention time point only were used to assess the effect of pen-level variables. Scatterplots were used to show the univariable relationship between quantitative pen-level variables (days in feeding, number of animals in the pen, liters of water per head) and within-pen prevalence. Logistic regression models were constructed to estimate the univariable effect of each pen-level variable. Continuous variables were modelled linearly. Diagnostic plots were used as described above.

Using the complete dataset, the proportions of fecal samples (and 95% confidence interval) that were positive for *E*. *coli* O157:H7 pre- and post-intervention, in control and intervention pens, were visualized graphically, by year. The within-pen prevalence values before and after the intervention were plotted, by intervention allocation group. Logistic regression models were fit to estimate the effect of the intervention on *E*. *coli* O157:H7 prevalence in fecal samples, as follows. The outcome of interest was individual-level prevalence of *E*. *coli* O157:H7. Fixed effects included (i) time-period (pre- and post-intervention); (ii) group allocation (intervention versus control); and (iii) the combined effect of time-period and group allocation (using an interaction term).

#### Multivariable analyses of associations between risk factors, intervention and proportion of fecal pats positive for *E*. *coli* O157:H7

A separate series of multivariable models was built for each weather variable. The choice of modelling the variable as a linear variable or as a categorical variable was made based on the univariable model diagnostics (described above) and the p-values. Firstly, pen and date were added into each univariable model as random effects. Likelihood ratio tests (LRT) were then conducted to assess the statistical significance of the association of the given weather variable with the prevalence of *E*. *coli* O157:H7 in the feces, after accounting for pen and date as random effects. If the p-value was <0.15, further multivariable analyses were carried out to assess potential confounding. Potential confounders of the association between each weather variable and the prevalence of *E*. *coli* O157:H7 in the feces were pre-selected based on the putative causal diagram (Fig 1) and the univariable p-value, and added into the model using a forward-selection stepwise procedure in increasing order of univariable p-values (including those with a p-value of <0.15). Potential confounders were retained in the model if the odds ratio (OR) for the effect of interest changed by >10%. In the final model for each weather variable, an interaction term between each statistically significant (p<0.05) variable and the variable of interest was added, one at a time. The LRT was conducted to assess the statistical significance of the interaction term. The interaction term was retained if the p-value was less than 0.1. Adjusted ORs, 95% confidence intervals and Wald-test p-values were presented based on the final model for each weather variable. Multivariable regression analyses were carried out according to the same protocol described above for pen-level variables.

Finally, multivariable regression analysis was carried out using the total dataset, to assess potential confounding of the observed association between group allocation (control vs. intervention), time-period (pre- or post-intervention) and the proportion of fecal pats positive for *E*. *coli* O157:H7. Pen and date were added into the model (described above) as random effects, to account for clustering within pens and within sampling dates. Potential confounders were identified based on the analysis of weather and pen-level variables described above, and were added into the model if they were statistically associated with the outcome and had a multivariable p-value <0.15. Forward selection was used. Variables were retained in the model if they resulted in a change in the OR for the effect of the intervention (the combined effect of time-period and group allocation) of >10%.

#### Sensitivity analysis

The analysis was repeated using data that excluded all pens that had a low level of water in the trough (<1.4 liters per head) at the start of intervention because there was an overlap between pre- and post-intervention water levels in intervention pens. This cut-off was chosen as after the intervention, the level of water in the trough in intervention pens did not exceed 1.4 liters per head. The analysis was also repeated including pens with a baseline within-pen prevalence of more than 0.1 to determine whether the effect of intervention is affected by the infection pressure within a pen. This cut-off was chosen arbitrarily.

#### Counts of coliforms and generic *E*. *coli* in the water troughs

The statistical analyses were repeated to explore the effect of explanatory variables on the log_10_ CFU/ml of coliforms and generic *E*. *coli* in the water trough as the outcome variables. Firstly, the Mann-Whitney U Test was used to compare the counts of coliforms and generic *E*. *coli* in the water troughs between intervention and control pens, before and after the interventions. Linear mixed effects models were then built, with date of sampling as a random effect. In order to check the assumptions of (1) homoscedasticity, (2) normal distribution of residuals and (3) linear relationship between exposure and outcome the following diagnostic plots were produced (respectively): (1) residuals versus predicted values of log_10_ CFU/ml counts of coliforms and generic *E*. *coli* in the water trough; (2) Q-Q plot and (3) residuals versus each predictor variable.

#### Association between generic *E*. *coli* in the water troughs and fecal prevalence of *E*. *coli* O157:H7

Mixed effect logistic regression models were fit to explore the strength of the association between log_10_ counts of coliforms or generic *E*. *coli* in the water troughs and *E*. *coli* O157:H7 prevalence in fecal samples. Log_10_ transformed counts of coliforms and *E*. *coli* were explored as linear variables. Pen and date were included as random effects. Potential confounders were identified based on the analysis of weather and pen-level variables described above, and were added into the model if they were statistically associated with the outcome and had a multivariable p-value < 0.15. Forward selection was used. Variables were retained in the model if they resulted in a change in the OR of the effect of interest of >10%. The assumption of a linear relationship between the log_10_ bacterial counts and the log odds of *E*. *coli* O157:H7 being present in the fecal pats was assessed using diagnostic plots.

#### Quantification of *E*. *coli* O157:H7 abundance in fecal samples using spiral plating

The median and range of counts of *E*. *coli* O157:H7 CFU/g in fecal samples were calculated from the spiral plate data. The differences between baseline and after the intervention, in control and intervention pens, were assessed using the Mann-Whitney U Test.

## Results

### Summary of samples obtained

As part of environmental screening before the trial, of 192 pens on the feedlot in 2014, 23 (12%) were identified as positive for *E*. *coli* O157:H7, 19 of which were included in the trial (4 pens with high days in feeding and scheduled for slaughter were excluded). In the environmental screening before the trial in 2015, of 203 pens, 35 (17%) were identified as positive, 16 of which were included in the trial. Out of the 16 pens, one intervention pen was lost to follow up (because the cattle were shipped for slaughter) after the pre-intervention sampling (and was excluded from the intervention trial analysis). As part of the two trials during the entire study period, 2,759 fecal samples were collected from 35 pens in total (a single fecal sample was lost in a pen in year 1), and 1,240 (45%) of the samples were collected in year 2. From the 35 pens, 69 water samples were collected during the entire study (one pen lost to follow up). *E*. *coli* O157:H7 was not detected in any of the water samples, however generic *E*. *coli* and coliforms were quantified.

### Results of environmental screening

There was no statistical association between whether *E*. *coli* O157:H7 was identified in a pen and either mean weight of animals, number of animals in the pen, or days in feeding (p>0.5; Mann-Whitney U test for continuous variables) (based on year 1 data; data not available for year 2).

### Descriptive analysis of the data

According to selective culture and latex agglutination testing, of the 2,759 fecal samples in total, 21.2% (95% CI 19.8–23.0) were positive for *E*. *coli* O157:H7 (weighted by the inverse sampling fraction by pen). Of the 69 water samples, *E*. *coli* were detected in 100%. The geometric mean *E*. *coli* count was 3.2 log_10_ CFU/ml (95% CI: 3.0–3.4). The geometric mean coliform count was 3.6 log_10_ CFU/ml (95% CI: 3.5–3.8). At the start of the trial, the days in feeding in each pen ranged between 37 and 355 days (median 223 days).

According to the spiral plating results conducted in year 2, only 32 fecal samples were identified with counts on the plate that were greater than zero (i.e., had high levels of *E*. *coli* O157:H7 considering the threshold of detection of 4 log_10_ CFU/g selected for interest in the distribution of super-shedders). Amongst these samples (control and intervention groups combined), the median count was approximately 10-fold higher after the intervention than at baseline (449,810 CFU/g versus 40,088 CFU/g); Mann-Whitney U Test; p 0.02). Unfortunately, the numbers of samples with non-zero counts were too low to assess the differences between control and intervention pens after the intervention.

**Fig 2:**
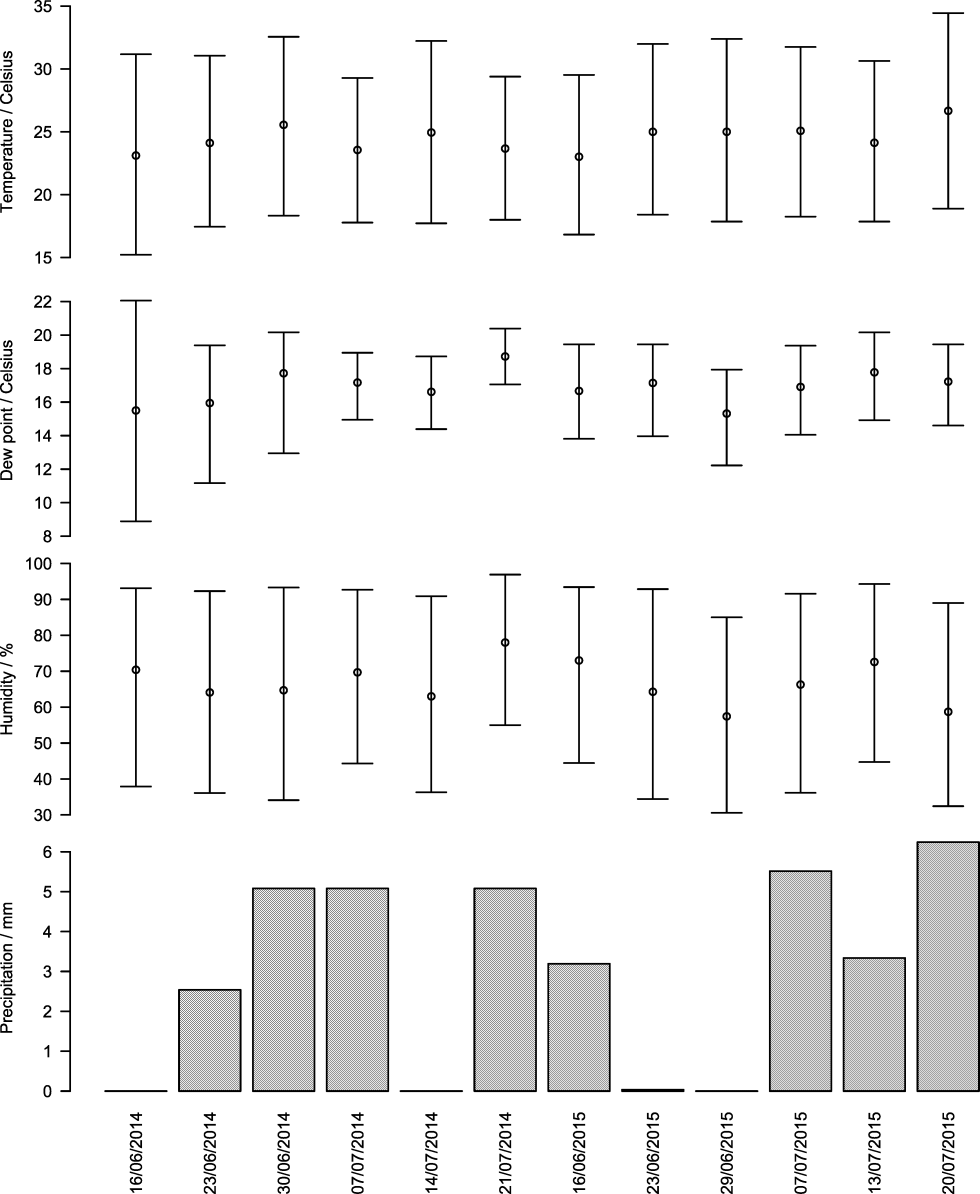
Mean temperature, dew-point, relative humidity and mean precipitation during the seven days preceding each sampling date. Points show 7-day mean of average daily values. Upper and lower horizontal lines show 7-day means of maximum and minimum daily values. Bars represent mean precipitation over the 7 days preceding the sampling date.

The pen population size ranged between 94 and 303 cattle (median 275). There were relatively minor variations in temperature, humidity, dew-point and precipitation between sampling dates (Fig 2). At baseline, intervention pens had a slightly smaller volume of water in the troughs than control pens (median approximately 1.5 liters per head; range 0.8–1.9), reducing to a median of 0.8 liters per head after the intervention (range 0.4–1.1). Control and intervention pens were similar with respect to days in feeding (which confirmed that blocking by days in feeding was successful) and pen population size.

### Effect of weather

**Fig 3:**
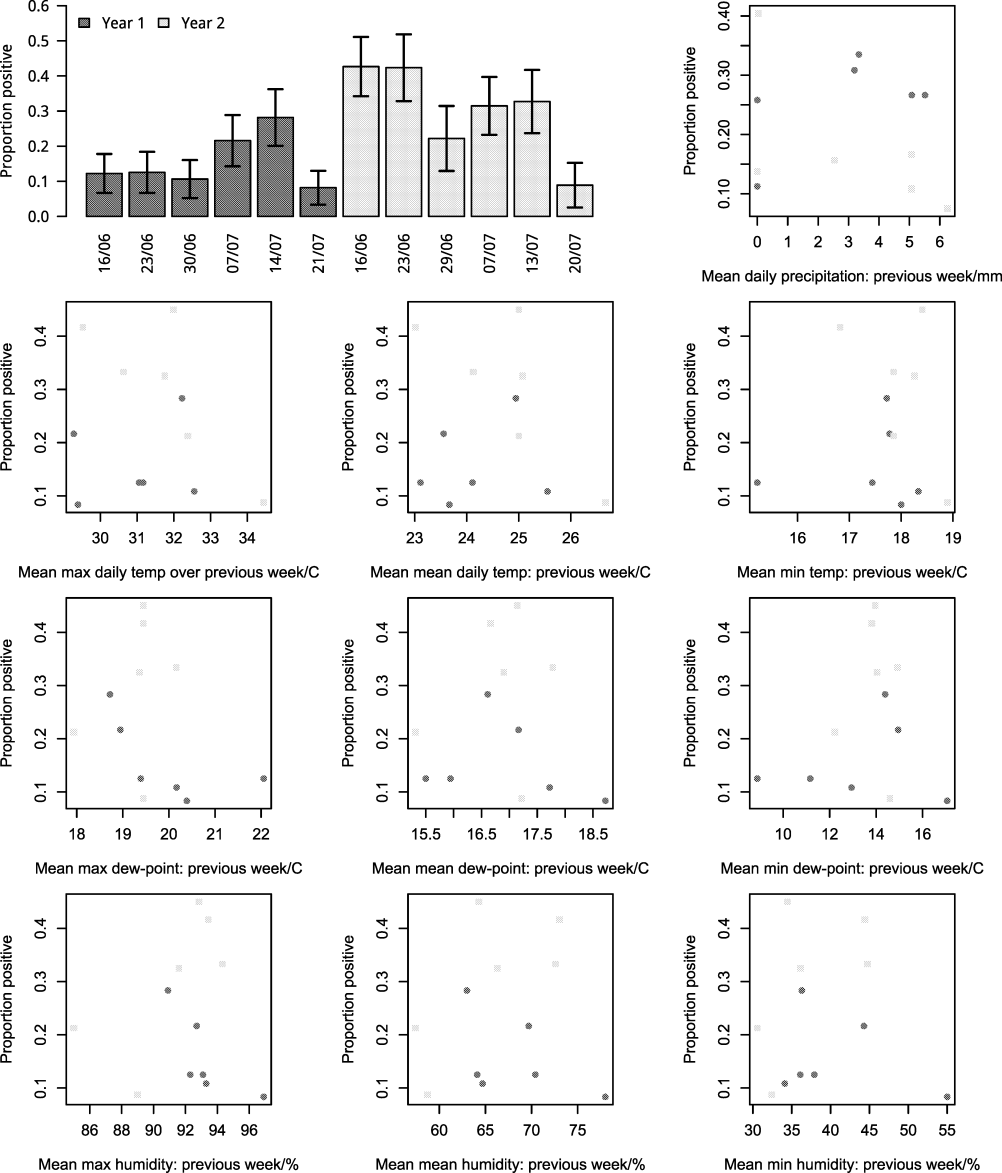
Proportion of fecal pats positive for *E*. *coli* O157:H7 by sampling date and weather conditions. Data shown from control pens only (number of fecal samples = 1360). Light gray indicates year 1 (2014). Dark gray indicates year 2 (2015). Bars indicate 95% confidence intervals.

A graphical representation of univariable associations between weather and proportion of samples testing positive are shown in Fig 3. After controlling for pen and date as random effects, the only weather variables that remained associated with the proportion of fecal pats positive for *E*. *coli* O157:H7 (number of samples = 1360) with a LRT p-value of < = 0.15 were: mean maximum dew-point over the previous week (as 3-level categorical indicator variables (each level having equal numbers of data points): p = 0.03; and as a linear variable: p = 0.03), mean average dew point over the previous week (as a linear variable: p = 0.11) and mean daily precipitation (as a linear variable: p = 0.15) (Table 1). After controlling for the effect of precipitation, mean maximum dew-point remained significant (p-value = 0.002) and a mean maximum dew-point of >20.2°C was associated with an approximate four-fold reduction in the odds of *E*. *coli* O157:H7 presence in fecal pats (OR = 0.3; 95% CI: 0.1–0.8), compared with a mean maximum dew-point of <19.4°C. After controlling for the effect of mean maximum dew-point, precipitation became strongly negatively associated with the odds of *E*. *coli* O157:H7 presence in fecal pats (p = 0.004), and an increase in one centimeter of precipitation was associated with a five-fold reduction in the odds of *E*. *coli* O157:H7 presence in fecal pats (OR = 0.2; 95% CI: 0.09–0.5). Mean average dew point became insignificant after controlling for confounders.

**Table 1:** Results of mixed effect logistic regression models to assess the association between weather variables and the log odds of *E*. *coli* O157:H7 presence in fecal pats, after adjusting for pen and date as random effects.

*Variable (unit)*	*Categories of predictor variable*	*Wald test p value*	*Odds Ratio*	*95% Confidence interval*
**mean minimum dew point in previous 7 days (°C)**	<13.9	baseline	baseline	baseline
	13.9–14.6	0.12	1.9	0.8–4.5
	>14.6	0.68	0.9	0.4–1.8
**mean average dew point in previous 7 days (°C)**	< 16.9	baseline	baseline	baseline
	16.9–63.0	0.54	1.3	0.6–2.8
	>63.0	0.10	0.5	0.2–1.1
**mean maximum dew point in previous 7 days (°C)**	< 19.4	baseline	baseline	baseline
	19.4–20.2	0.46	1.3	0.6–2.7
	>20.2	0.01	0.3	0.1–0.8
**mean maximum dew point in previous 7 days (°C)**	modelled as a linear variable	0.01	0.70 (per °C)	0.53–0.91
**mean daily precipitation (mm)**	modelled as a linear variable	0.15	0.90 (per mm)	0.78–1.0

Analysis was conducted using control pen data only. Only results with a p-value of < = 0.15 are shown. Variables with a p-value of > 0.15 (not shown) included: precipitation in previous 7 days; mean minimum temperature in previous 7 days; mean average temperature in previous 7 days; mean maximum temperature in previous 7 days; mean minimum humidity in previous 7 days; mean average humidity in previous 7 days; mean maximum humidity in previous 7 days.

### Effect of pen-level variables

**Fig 4:**
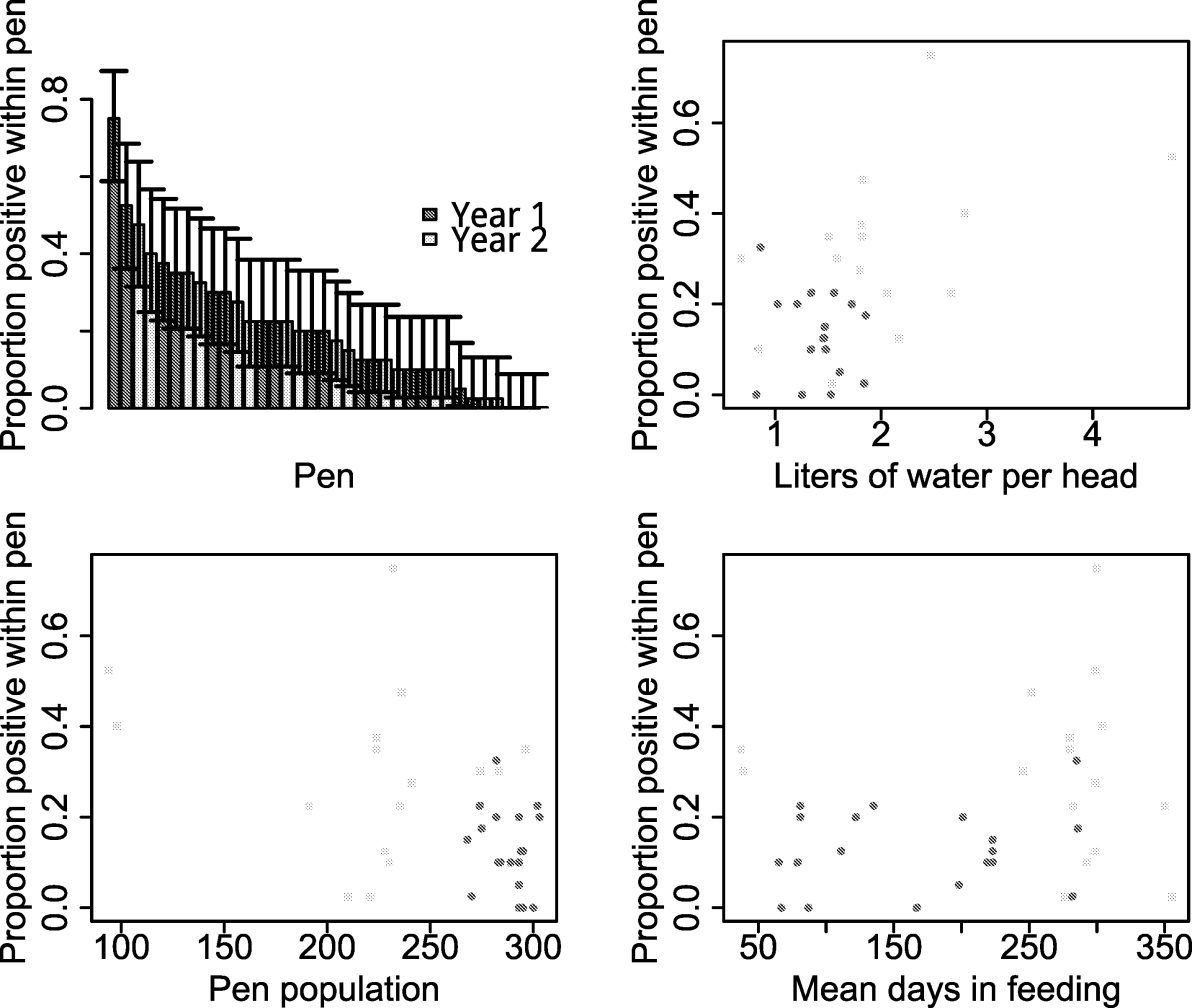
Proportion of fecal pats positive for *E*. *coli* O157:H7 by pen, and pen-level variables. Data shown from pre-intervention time-point only (number of fecal samples = 1400). Light gray indicates year 1 (2014). Dark gray indicates year 2 (2015). Bars indicate 95% confidence intervals. Note: clustering of pens by sampling date is not shown.

Although all pen-level variables (liters of water per head, days in feeding and pen population size) were each individually associated with the proportion of fecal pats positive for *E*. *coli* O157:H7 (Fig 4; number of fecal samples = 1400), none remained significant after controlling for pen and date as random effects. The Wald test results were as follows: pen population p = 0.23; liters of water per head p = 0.11; mean days in feeding p = 0.32. There was no change in the results when other potential confounders were included as fixed effects (LRT: p>0.15).

### Effect of intervention

**Fig 5:**
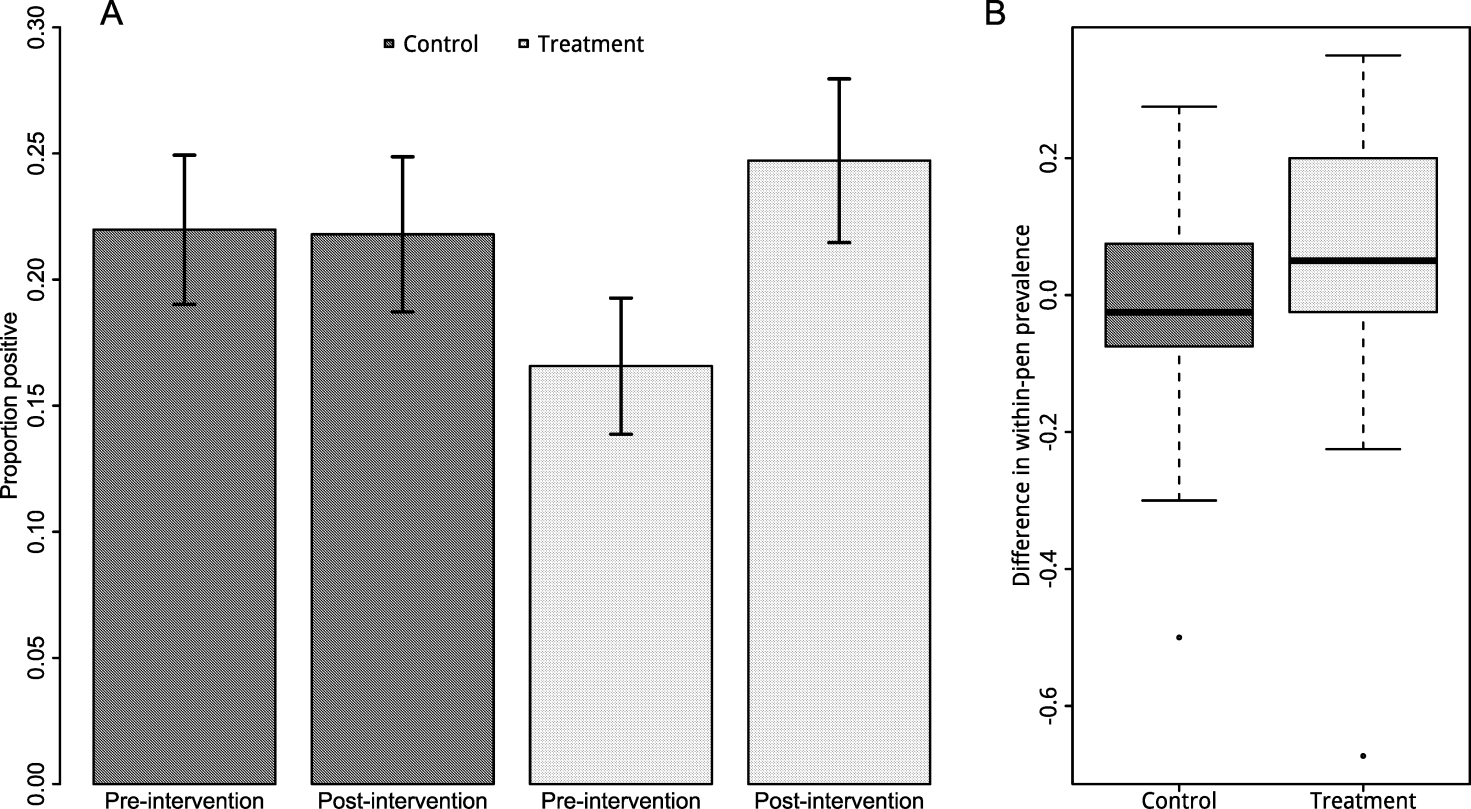
The change in the proportion of cattle fecal pats positive for *E*. *coli* O157:H7 over time. (A) The proportion of fecal pats positive for *E*. *coli* O157:H7 by control and intervention groups, pre- and post-intervention. Bars indicate 95% confidence interval after adjusting the standard errors for clustering within pens. (Number of fecal samples = 2719) (B) Box-plots of the (unadjusted) change in within-pen prevalence of positive fecal pats within each pen (post- minus pre-intervention prevalence). (Number of fecal samples = 2719).

There was an increase in the overall prevalence of *E*. *coli* O157:H7 amongst intervention pens three weeks after reducing the level of water in the trough, whereas there was no statistically significant difference in the overall prevalence of *E*. *coli* O157:H7 amongst control pens before and after the intervention (Fig 5). Fecal pats in the intervention pens experienced 1.7 times the odds of being positive to *E*. *coli* O157:H7 after controlling for the underlying temporal trend and differences between *E*. *coli* O157:H7 presence in feces in control and intervention pens at the pre-intervention time point (95% CI: 1.4–2.1; LRT: p = 0.003; number of fecal samples = 2719).

After controlling for pen and date as random effects, the combined effects of intervention and time remained significant (OR = 1.6; 95% CI: 1.2–2.0; LRT: p = 0.02; number of fecal samples = 2719). The effect of the intervention was not confounded by maximum average daily dew-point or total daily precipitation over the previous week. (Both variables were significant in the risk factor analyses based on p<0.15 as detailed above.)

### Sensitivity analysis

When the data were restricted to pens with an initial water-trough level of >1.4 liters per head, the crude OR for the effect of the intervention increased to 2.3 (95% CI: 1.8–2.7; p = 0.0003; number of fecal samples = 1960). After adjusting for pen and date as random effects, the adjusted OR was 1.9 (95% CI: 1.1–3.1; p = 0.01; number of fecal samples = 1960). When the data were restricted to pens with an initial within-pen prevalence of >0.1, the results were not appreciably affected although the adjusted p-value was 0.07 (number of fecal samples = 560).

### Generic *E*. *coli* and coliform counts in water troughs

None of the pen-level variables (liters of water per head, days in feeding and pen population size) were linearly associated with the counts of generic *E*. *coli* or coliforms in the water troughs after accounting for date as a random effect (F test; p >0.10; number of water samples = 69). Diagnostic plots did not suggest evidence of a non-linear relationship. Amongst the weather variables, none were associated with the counts of *E*. *coli* or coliforms in the water troughs (F test; p > 0.05; number of water samples = 69).

There were no significant differences between log_10_ CFU/g generic *E*. *coli* or coliform counts between intervention and control groups (Mann-Whitney U Test; p = 0.64; number of water samples = 68), before and after the intervention in the control group (Mann-Whitney U Test; p = 0.96; number of water samples = 34) or in the intervention group (Mann-Whitney U Test; p = 0.89; number of water samples = 34). The addition of date as a random effect into the linear models did not appreciably affect the results.

### Association between generic *E*. *coli* in the water troughs and fecal prevalence of *E*. *coli* O157:H7

After controlling for sampling date and pen as random effects, there was no evidence of a linear association between counts of generic *E*. *coli* (LRT: p = 0.31) or coliforms (LRT: p = 0.08) in water troughs (number of water samples = 69) and the log odds of *E*. *coli* O157:H7 in fecal pats (number of fecal samples = 2759). Diagnostic plots provided no evidence suggesting a non-linear relationship.

## Discussion

This intervention trial provided evidence that decreasing the level of water in troughs in feedlot pens that are already contaminated with *E*. *coli* O157:H7 may modestly increase the proportion of fecal pats that are positive for *E*. *coli* O157:H7. To our knowledge, this is the first time that decreasing water levels in troughs has been shown to be associated with an increased risk of *E*. *coli* O157:H7 fecal shedding, and the result was surprising given that previous modeling suggested that the effect would be in the opposite direction [24,31].

Since pens were allocated to intervention or control status using systematic random sampling, the risk of bias leading to a false finding of an effect was minimized. We found no evidence that the observed relationship between water-level and prevalence of *E*. *coli* O157:H7 in fecal pats was confounded by weather or pen population size (the potential effect of days in feeding was controlled through the study design by blocking). We cannot rule out the possibility of an unmeasured confounder, such as a spatially-related variable, e.g., topology or microenvironments which may vary between pens, and could be related to moisture and survival of *E*. *coli* O157:H7 in the fecal pats. However, mapping of the positive and negative pens in both years did not reveal a spatial pattern. Furthermore, the finding was consistent in years one and two, despite the fact that there was an overall trend of increasing prevalence in year one, from the beginning to the end of the trial, and an overall trend of decreasing prevalence in year two. Further, the magnitude of effect was greater for pens that had a high initial water-trough level, which is consistent with what would be expected if this was a true causal effect.

Although changing the water-trough level appeared to affect fecal prevalence of *E*. *coli* O157:H7, no *E*. *coli* O157:H7 was identified in any of the water troughs, and we did not detect an association between generic *E*. *coli* or coliform counts in the water troughs and either fecal prevalence of *E*. *coli* O157:H7 or the intervention. In view of these water results we can only speculate on the mechanism of action. The apparent absence of *E*. *coli* O157:H7 from the water troughs may be explained by the relatively low sensitivity of the test for the volume of water tested. The detection limit for *E*. *coli* O157:H7 in water samples was 1 CFU/ 100 ml meaning that concentrations below that threshold could have been missed.

The purpose of enumerating the generic *E*. *coli* and coliforms in water troughs was to use them as indicators of fecal contamination that may help elucidate the effect of the implemented intervention on the bacterial dynamics in a trough. It is possible that re-testing water at 3-week intervals was insufficient to capture the causative relationship, if real, between water contamination and animal colonization. Cattle colonization has been reported to last up to 3 weeks [32] meaning that even cattle that became colonized soon after the intervention was implemented could still be colonized and observed at the time of re-testing 3 weeks later. Alternatively, the level of water contamination may have varied much more rapidly even within a day due to the stochastic nature of contamination events and rapid water turnover rate. Water turnover rate is the time it takes for cattle to drink the volume of water equal to the total volume of the water trough; it is calculated by dividing the volume of water with the water flow rate. In a pen with 200 cattle each drinking 50 liters of water per day [40] from a water trough that is automatically refilled to the capacity of 400 liters, the water turnover rate would be 400/(200*50) = 0.04 days or 0.96 hours, meaning that cattle would drink approximately the volume of the trough every hour or 24 times per day. Reducing the water volume to a half in the intervention pens reduced that time to a half, meaning that fluctuations in contamination level would occur at a faster rate in those pens. Using a simple mathematical model of bacterial contamination in an automatically refilled water trough (not presented), it can be shown that under the assumptions of constant rates of water contamination and drinking, homogenous mixing within the trough and no growth or decay of bacteria in water, the concentration of bacteria would approach the same equilibrium level regardless of water volume, but that the equilibrium level would be approached faster in the intervention compared to control pens. At timescales relevant to cattle exposure to contaminated water through drinking, the water contamination rate is unlikely to be constant. The events that lead to contamination of water are stochastic in their timing and variable in the amount of introduced bacteria. Likewise, cattle drink water at a variable rate during a 24 -hour period, resulting in fluctuations in the concentration of bacteria. Assume two troughs refilled to the levels of 400 liters and 200 liters, respectively, in otherwise identical control and intervention pens, and instantaneous and homogeneous dispersal of bacteria after their introduction into water. After a single introduction of the same amount of bacteria into each of the two troughs, the concentration of bacteria would be twice as large in the intervention compared to the control trough, because the bacteria would be more diluted in the larger volume of water, but the bacteria would be eliminated faster from the intervention compared to the control trough through cattle drinking. Therefore, reducing the volume of water in intervention pens may have had a short-term effect on the concentration of bacteria in the trough post each contamination event that resulted in an increased dose of bacteria being consumed by cattle. While this mechanism of action may seem feasible, to capture it empirically would require more frequent sampling of water and cattle than was feasible in this study.

As another possible explanation of the mechanism of action, reducing the water level could have caused debris at the bottom of the water troughs to be disturbed more easily and frequently, causing the cattle to consume more *E*. *coli* O157:H7. The debris with *E*. *coli* O157:H7 may occur as part of the environmental contamination with feces contaminated with *E*. *coli* O157:H7, which has been shown to be important in the spread of *E*. *coli* O157:H7 [32,41]. Alternatively and unlikely, reducing the water level could have changed the behavior of the cattle, for example causing them to drink more or less frequently, which could have some effect on the probability of colonization of the intestinal tract. It has been shown that trough height can affect cattle drinking behavior, although trough depth did not seem to have an effect [42]. However, predictors of colonization of the intestinal tract are currently not well understood, so this is only speculative [43].

As expected, there was clustering of *E*. *coli* O157:H7 shedding within pens and within sampling dates. Other studies have observed similar variability in fecal shedding between pens and within pens over time [22]. Despite this between-pen and between-sampling date variability, the only predictors that we found to be significantly associated with fecal prevalence were a high mean maximum dew-point in the previous seven days and increased rainfall in the previous seven days, both of which were negatively associated with fecal prevalence. Together, these findings suggest that higher moisture was associated with lower numbers of *E*. *coli* O157:H7 in the environment. The relationship between *E*. *coli* O157:H7 survival and environmental conditions is complex; dry conditions can decrease survival of *E*. *coli* in the environment through water stress, whereas high levels of water can decrease survival by producing anoxic conditions [44]. The apparent effects of precipitation and dew point in previous studies have been relatively small and inconclusive [45–47]. One study of four calves reported a reduced self- and allo-grooming behavior during wet periods, and this could in theory reduce spread of *E*. *coli* O157:H7 through licking of contaminated hides [48]. Finally, there were no significant predictors of the probability of a pen having positive *E*. *coli* O157:H7 status according to the environmental screening. This may be in part due to the high level of variability in *E*. *coli* O157:H7 prevalence over time, which is not captured by pen-level variables.

This study has a few important limitations. The latex agglutination test without PCR confirmation can in some cases detect *E*. *coli* other than *E*. *coli* O157:H7, and this could have resulted in an over-estimation of prevalence. However, this non-differential misclassification bias would result in an under-estimation of any effect of the intervention as it would apply to control and intervention groups equally, meaning that the true association between the intervention and *E*. *coli* O157:H7 faecal shedding could have been even stronger [49]. Unfortunately, the change in feedlot management in year 2 made some analyses impossible because of unavailable data. Also, the concurrent commercial trials in year 2 restricted our ability to fully randomize pens, which could have introduced confounding. The study showed the effect of reduced water level on *E*. *coli* O157:H7 shedding prevalence in only one feedlot over a relatively short time-period: three weeks. Furthermore, the results may not be reproducible in different climatic conditions. However, repeatability of the findings in year 1 and 2 contribute to the evidence that a true effect was observed. *E*. *coli* O157:H7 shedding within an individual animal is highly variable and dependent on complex interactions between the cattle and the environment, which may have reduced the power of the study to observe more subtle effects.

In conclusion, despite the limitations, this study provides evidence that keeping water levels in the troughs high could prevent potential increases in prevalence of *E*. *coli* O157:H7 fecal shedding. Further, this suggests a novel control strategy for reduction of *E*. *coli* O157:H7 at the pre-harvest level. However, increasing the water-level in troughs was not directly tested and should therefore be investigated with longer-term trials in different settings before implementation of the strategy.

## Supporting information

S1 Table Counts of *E*. *coli* O157:H7 in fecal samples based on spiral plating. Shown are only samples with counts >0 CFU/g of feces. Notations: n = number of samples; med = median, min = minimum and max = maximum count.
(PDF)

S1 File Exposure and outcome variables for each fecal pat included in the intervention trial. (CSV)

S2 File Description of variables in S1 File. (PDF)
