# Supplementary material for: The prevalence of Escherichia coli O157:H7 fecal shedding in feedlot pens is affected by the water-to-cattle ratio: A randomized controlled trial
Source: PLoS One. 2018 Feb 7;13(2):e0192149. doi: 10.1371/journal.pone.0192149 (PMC5802916; doi:10.1371/journal.pone.0192149)
Supplement: S1 Table — Shown are only samples with counts >0 CFU/g of feces. Notations: n = number of samples; med = median, min = minimum and max = maximum count. (PDF) [file pone.0192149.s001.pdf]

Table. Counts of *E. coli* O157:H7 in fecal samples from spiral plating. Shown are only samples with counts >0 CFU/g of feces. Notations: n=number of samples; med=median, min=minimum and max=maximum count.

	Control Pens (n = 13)	Intervention Pens (n = 19)	All Pens (n = 32)
Pre-intervention (n = 25)	med = 44970<br>min = 10220<br>max = 1410420	med = 29630<br>min = 10220<br>max = 2290180	med = 40880<br>min = 10220<br>max = 2290190
Post-intervention (n = 7)	med = 95900<br>min = 20450<br>max = 449800	med = 531590<br>min = 134940<br>max = 940510	med = 449810<br>min = 20450<br>max = 940510
All samples (n = 32)	med = 44970<br>min = 10220<br>max = 1410430	med = 76650<br>min = 10220<br>max = 2290190	med = 60810<br>min = 10220<br>max = 2290180
