# Supplementary material for: The prevalence of Escherichia coli O157:H7 fecal shedding in feedlot pens is affected by the water-to-cattle ratio: A randomized controlled trial
Source: PLoS One. 2018 Feb 7;13(2):e0192149. doi: 10.1371/journal.pone.0192149 (PMC5802916; doi:10.1371/journal.pone.0192149)
Supplement: S2 File — (PDF) [file pone.0192149.s003.pdf]

Table. Description of variables in S1 File

**Variable name**	**Variable description**
sample_ID	Unique ID for each sample
id	ID number for pen
year	Year of study (1 or 2)
date	Date of sampling/testing
time	Pre- (0) or post- (1) intervention
timeL	Pre- (B) or post- (A) intervention
block	Block=stratum by 'days in feeding'. In Year 1: block I (31-67 days), block II (77-178 days) and 6 pens in block III (189-265 days)
pen	2nd ID number for pen
cohort	Cohort number (relates to date at which intervention began)
cohortY	Cohort number plus year number
headsE	Number of animals in pen at the time of environmental sampling
difE	'Days in feeding' at the time of environmental sampling (= days since animals arrived on the feedlot)
weightE	Mean weight in pounds at time of environmental sampling
code	3rd ID code for pen (A, B, C…..PP)
group	Intervention group (T) or Control group (C)
timegroup	Time (pre- or post) plus group (treatment or control)
heads0	Number of animals in pen at the beginning of the trial
dif	'Days in feeding' (= days since animals arrived on the feedlot)
volume0	Volume of water in the tank at the beginning of the trial in cubic inches
gallons	Volume of water in the tank at the beginning of the trial in Gallons [Fluid, US]
GALperHEAD	Volume of water in the tank in Gallons [Fluid, US] divided by the number of animals in the pen.
feedlotarea	Three different areas on the feedlot (A, B or C)
feces	Positive for *E. coli* O157:H7 (=1) or negative (=0)
f.count	Fecal count of *E. coli* per ml of solution. (= fecal count per 0.1g of feces)
f.countLog10	Log of f.count
Coliforms	Count of coliforms per ml of water from water trough.
Ecoli	Count of generic *E. coli* per ml of water from water trough.
tx7F	Maximum temperature over the previous 7 days in Fahrenheit
ta7	Mean temperature over the previous 7 days in Fahrenheit
tn7F	Minimum temperature over the previous 7 days in Fahrenheit
dpx7F	Maximum dew point over the previous 7 days in Fahrenheit
dpa7F	Mean dew point over the previous 7 days in Fahrenheit
dpn7F	Minimum dew point over the previous 7 days in Fahrenheit
hx7	Maximum humidity over the previous 7 days in Fahrenheit

ha7	Mean humidity over the previous 7 days in Fahrenheit
hn7	Minimum humidity over the previous 7 days in Fahrenheit
wsx7MPH	Maximum wind speed over the previous 7 days in Fahrenheit
wsa7MPH	Mean wind speed over the previous 7 days in Fahrenheit
gsx7MPH	Maximum gust speed over the previous 7 days in Fahrenheit
p7In	Mean daily precipitation in inches over the previous 7 days
CloudCover	Numeric variable representing level of cloud cover.
Events	Weather events e.g. rain, thunder. Coded numerically.
WindDirDegrees	Direction of wind in degrees from North.
